# Clinical subphenotypes and molecular endotypes in sepsis: toward an integrated and dynamic framework

**DOI:** 10.1016/j.aicoj.2026.100060

**Published:** 2026-04-06

**Authors:** Guiyu Zhang, Xiaojing Wu, Songqiao Liu, Hongyang Xu, Chenglong Cai, Jiawei Liu, Siqi Liu, Pufeng Wang, Jianfeng Xie

**Affiliations:** a Jiangsu Provincial Key Laboratory of Critical Care Medicine, Department of Critical Care Medicine, Zhongda Hospital, School of Medicine, Southeast University, Nanjing, Jiangsu, China; b The First People's Hospital of Lianyungang, The Affiliated Lianyungang Hospital of Xuzhou Medical University, Lianyungang 222000, Jiangsu, China; c Department of Critical Care Medicine, The Affiliated Wuxi People's Hospital of Nanjing Medical University, Wuxi People's Hospital, Wuxi Medical Center, Nanjing Medical University, Wuxi, 214000, China

**Keywords:** Sepsis, Subphenotype, Endotype, Longitudinal analysis, Precision medicine

## Abstract

Sepsis is a highly heterogeneous and life-threatening syndrome associated with high morbidity and mortality worldwide. The repeated failure of large randomized trials underscores the urgent need for precise patient stratification. Recent advances in machine learning and multi-omics technologies have facilitated the identification of distinct clinical subphenotypes and molecular endotypes. Clinical subphenotypes, typically derived from routinely available clinical variables and circulating biomarkers, reflect aggregated downstream manifestations of underlying biological processes; however, the absence of clearly identifiable pathobiological mechanisms specific to each subgroup may limit their utility as actionable treatable traits. In contrast, molecular endotyping leverages multi-omics data to elucidate the pathophysiological drivers of sepsis, offering a foundation for mechanism-based interventions. However, most endotypes remain insufficiently actionable for individualized treatment decisions at the bedside. Furthermore, existing classification frameworks rely predominantly on static assessments, which do not adequately reflect the dynamic evolution of sepsis pathophysiology. Increasing evidence underscores that sepsis is inherently dynamic, with immune responses, metabolic states, and organ dysfunction fluctuating over time. Integrating longitudinal clinical and molecular data to capture the temporal evolution of host responses and organ dysfunction through dynamic subtyping offers a promising approach to optimize patient stratification. In this narrative review, we summarize recent advances in static and dynamic subphenotyping, discuss omics-derived endotypes, and outline strategies to integrate these dimensions into clinically actionable frameworks for precision medicine in sepsis.

## Introduction

Sepsis, a life-threatening organ dysfunction resulting from a dysregulated host response to infection, remains a major global health burden with an estimated 48.9 million cases and 11 million deaths in 2017 [1,2]. Despite extensive basic research and preclinical trials in recent years identifying multiple therapeutic targets, most interventions have failed in large-scale clinical trials, and current treatment still primarily relies on early antimicrobial therapy, source control, and supportive care, with limited mechanism-based precision therapies [3].

The high failure rate of sepsis clinical trials is attributed in large part to the biological and clinical heterogeneity of sepsis populations [4]. Increasing evidence suggests that sepsis patients exhibit significant differences in pathogen types, immune responses, metabolic states, and genetic background, which not only affect clinical features and prognosis but also directly determine their response to specific therapeutic interventions. In conventional trials, the “average treatment effect” often masks subgroup-specific responses, causing treatment measures that might benefit certain subgroups to fail to demonstrate their advantages and, in some cases, to be ineffective or even potentially harmful to other patients, which has become one of the major obstacles to the development of precision medicine for sepsis.

**Fig. 1:**
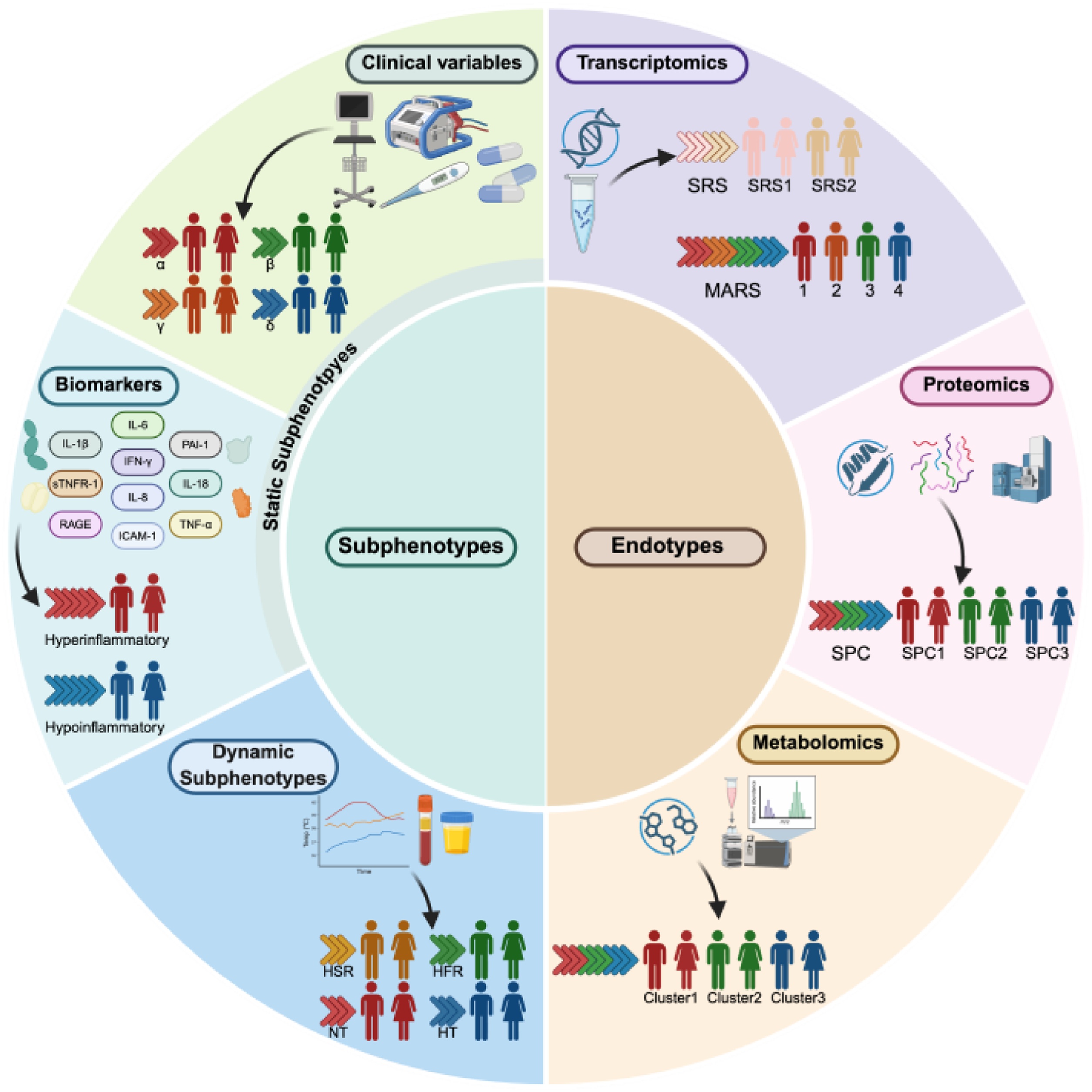
Overview of current approaches to sepsis subtyping. This figure illustrates how machine learning applied to clinical features, biomarkers, and multi-omics data enables the identification of distinct subphenotypes and endotypes, thereby providing a mechanistic foundation for informing targeted therapeutic strategies. Figure created with BioRender.com

Recently, advances in machine learning algorithms and multi-omics technologies have provided new tools for studying patient heterogeneity. Subtyping frameworks have been successfully applied in oncology and asthma, laying the groundwork for sepsis subtyping research [[5], [6], [7]]. Increasing efforts are being made to classify sepsis into subphenotypes and endotypes to uncover underlying biological differences and guide precision therapy (Fig. 1).

While these approaches have provided important insights, most frameworks treat subgroups as static and mutually exclusive. In reality, host responses in sepsis are dynamic, evolving over time. Integrative strategies that combine clinical variables, circulating biomarkers, and high-dimensional omics data within a longitudinal framework are required. The goal of such integration is to identify biologically coherent, temporally adaptive disease states that can guide differential treatment strategies. By shifting from average treatment effects to state-specific, time-sensitive interventions, integrative models may overcome limitations that have historically constrained sepsis trials. In this review, we summarize static and dynamic clinical subphenotypes, omics-derived endotypes, and explore integrative, longitudinal frameworks, highlighting implications for future trial design and personalized treatment.

## Literature search and conceptual framework

We searched PubMed for studies published up to February 2026 to identify key research on sepsis subtypes. Literature for this Review was obtained using a combination of MeSH terms and free-text keywords. The core search terms included (“Sepsis” OR “Septic Shock” OR “Bacteremia”) AND (“Subtype” OR “Subphenotype” OR “Endotype”). Additional topic-specific terms were applied as needed to broaden coverage of mechanistic, clinical, and translational aspects of sepsis heterogeneity. This review was designed as a narrative synthesis supported by targeted literature searches and expert judgment, rather than as a formal systematic review. The final body of references was curated according to their relevance to the review’s aims and refined through consensus among the authors, rather than through predefined systematic screening protocols. Articles identified through database searches and those cited in the reference lists of relevant publications were all considered for inclusion.

To ensure conceptual precision, we applied a consensus-based phenotyping framework grounded in the contemporary literature [8,9]. A phenotype is defined as a clinically observable set of traits arising from the interaction between genotype and environmental exposures, such as sepsis or ARDS. A subphenotype represents a distinct subgroup that is reliably discriminated through data-driven assessments of multidimensional properties and is reproducible across populations. An endotype is a subphenotype with distinct functional or pathobiological mechanism with potential implications for heterogeneity in treatment response.

## Subphenotypes of sepsis

Currently, multiple subphenotypes of sepsis and other critical illnesses have been identified through the integration of diverse data types, including clinical characteristics and biomarkers (Table 1). Among these, certain subphenotypes have not only been consistently replicated across independent cohorts, but also used to assess patients’ responses to specific therapeutic interventions, thereby providing critical insights into the advancement of precision medicine.

**Table 1:** Pivotal studies of subphenotypes in sepsis.

Subphenotypes	N	Clustering method	Handling of dropouts	Characteristics	Differential treatment response	Classifier
Clinical Static Subphenotypes						
SENCA (α, β, γ, δ) [10]	20,189	Consensus k-means clustering	Multiple imputation with chained equations (MICE)	α: fewer abnormal laboratory values and minimal organ dysfunction β: older, more chronic illness, and renal dysfunction γ: high inflammation (elevated WBC, bands, ESR, CRP), hypoalbuminemia, and fever δ: elevated lactate, transaminases, and hypotension	γ: benefited from rhAPC and Xuebijing therapies δ: experienced increased harm from early goal-directed therapy; but benefited from vilobelimab and Xuebijing	None reported
COVIDICU1, COVIDICU2, COVIDICU3 [14]	13,279	Consensus k-means clustering	Chained random forests	COVIDICU1: younger, with the lowest APACHE scores, highest BMI, lowest PaO2/FiO2 ratio COVIDICU2: lowest BMI and glucose, highest PaO2/FiO2 ratio, and fewest comorbidities COVIDICU3: eldest, with the most comorbidities, the highest APACHE scores, acute kidney injury and metabolic dysregulations	COVIDICU1 and COVIDICU2: experienced increased mortality from corticosteroid therapy COVIDICU3: showed no harm effect from corticosteroid therapy	None reported
Biomarker-driven Static Subphenotypes						
Hypoinflammatory, Hyperinflammatory [18]	745	Latent class analysis	Not addressed	Hypoinflammatory: lower cytokines, higher SBP, higher platelets, more trauma, aspiration, or pneumonia-related ARDS Hyperinflammatory: higher IL-6, IL-8, sTNFR-1, PAI-1, Ang-2, RAGE, ICAM-1, IL-1β, TNF-α, IL-2, and IL-4, elevated creatinine and bilirubin, bicarbonate and protein C, more sepsis-related ARDS	Hyperinflammatory: benefited from corticosteroid and activated protein C; demonstrated improved survival and transition toward the Hypoinflammatory state with corticosteroid therapy	parsimonious classifier models (PCMs): three-variable (IL-8, bicarbonate, and protein C) and four-variable (IL-8, bicarbonate, protein C, and vasopressor use) models clinical-data-only classifier model (CCM)
Dynamic Subphenotypes						
Group 1−4 [35]	12,413	Group-based trajectory model	Not addressed; earliest measurement selected within each 1 -h window	Group 1: hyperthermic, slow resolvers Group 2: hyperthermic, fast resolvers Group 3: normothermic Group 4: hypothermic	Not tested	None reported
Group A–D [39]	20,729 (12,473 derivation, 8,256 validation)	Group-based trajectory model	Not addressed	Group A: hyperthermic, tachycardic, tachypneic and hypotensive Group B: hyperthermic, tachycardic, tachypneic (not as pronounced as Group A) and hypertensive Group C: lower temperatures, heart rates, respiratory rates, and normotensive Group D: lower temperatures, heart rates, respiratory rates, and hypotensive	Group A and B: experienced increased mortality with tocilizumab therapy Group C: showed improved survival with tocilizumab therapy Group D: showed lower 30-day mortality with balanced crystalloids compared to saline	None reported

### Static subphenotypes

Static subphenotypes of sepsis refer to patient classifications derived from information from a single time point, typically clinical data or biomarker levels at admission, which are commonly applied in early stratification or intervention. Compared with dynamic subphenotype analyses, they are simpler, more accessible, and highly interpretable, thus widely adopted in clinical research.

#### Clinical static subphenotypes

Clinical static subphenotypes of sepsis are defined as patient clusters identified through unsupervised clustering algorithms or latent variable models using admission data such as vital signs, comorbidities, and laboratory results. Relying on routinely collected and clinically familiar data, they offer strong interpretability and high feasibility for bedside implementation. In recent years, multiple models have been developed using only routinely available variables, underscoring their potential for rapid deployment in diverse settings.

A multicenter retrospective analysis using 29 clinical variables identified four static clinical subphenotypes of sepsis that differed in prevalence, organ dysfunction patterns, and mortality [10]. The α subphenotype represented milder disease, the β subphenotype was associated with older age and comorbidities [11], the γ subphenotype reflected hyperinflammation, and the δ subphenotype exhibited severe circulatory and metabolic dysfunction with the highest mortality. Trial simulations suggested increasing harm from early goal-directed therapy (EGDT) with higher proportions of δ patients. Consistently, treatment effects differed across subphenotypes: recombinant human activated protein C benefited the γ subphenotype [12], vilobelimab improved outcomes mainly in the δ subphenotype [13], and Xuebijing injection reduced mortality in the γ and δ subphenotype.

However, the reproducibility of existing static subphenotype frameworks remains inconsistent across studies. Several recent analyses have derived clinical static subphenotypes using routinely available variables in sepsis and septic shock, supporting their potential utility for rapid risk stratification and early clinical decision-making, particularly in resource-limited settings [14,15]. Nevertheless, several limitations remain. Current subphenotype derivation relies primarily on routinely collected electronic health records (EHR) and may change when additional biological or pathogen-related data are incorporated. For example, in the SENECA framework, nearly half of patients were reassigned to different subphenotypes after microbiological data were incorporated, and external validation across cohorts has remained limited [12,16]. In addition, clustering results may be influenced by methodological assumptions, data completeness, and cohort heterogeneity. Clinical subphenotypes may provide a practical framework for early risk stratification, although their robustness remains sensitive to cohort composition and methodological assumptions. Further research is needed to enhance the generalizability and reproducibility across diverse clinical settings.

#### Biomarker-driven static subphenotypes

Biomarker-driven static subphenotypes of sepsis are constructed using biomarker data such as inflammatory cytokines and immune-related proteins at a single time point to uncover the mechanistic heterogeneity in the early stages of the disease. Clustering analyses identify patient subgroups with distinct immune profiles, elucidating the critical role of immune dysregulation in the pathogenesis and progression of sepsis. Moreover, these subphenotypes hold translational potential for prognostic prediction and immunomodulatory therapies, serving as a key bridge toward the advancement of precision medicine.

Based on integrated clinical and biomarker data, subphenotypes characterized as Hyperinflammatory and Hypoinflammatory have been described in sepsis cohorts [17,18]. The Hyperinflammatory subphenotype is characterized by higher levels of inflammatory and endothelial biomarkers, more severe organ dysfunction, and poorer clinical outcomes. To facilitate identification, parsimonious biomarker models, clinical classifiers, and AI-based classifiers have been established [[19], [20], [21], [22]], alongside efforts to develop point-of-care testing (POCT) for timely clinical application (NCT04009330) [23]. Treatment responses differ across inflammatory subphenotypes, with Hyperinflammatory patients benefiting from corticosteroids and activated protein C, whereas rosuvastatin has shown no consistent subphenotype-specific benefit [17,18,24]. Complementary biomarker-centric analyses have identified two related subphenotypes, Uninflamed and Reactive, which broadly correspond to Hypoinflammatory and Hyperinflammatory profiles, respectively [25]. The Reactive group shows greater biomarker activation, increased disease severity, and enrichment of oxidative phosphorylation and cholesterol metabolism pathways [26].

Building upon these biomarker-driven classification frameworks, recent studies have further defined two clinically actionable, biomarker-driven static subphenotypes: Macrophage Activation-Like Syndrome (MALS), Intermediate state and Sepsis-Induced Immunoparalysis (SII), with remaining patients representing an intermediate immune state. These subphenotypes are defined by blood ferritin levels and HLA-DR expression on CD45/CD14 monocytes, with MALS reflecting hyperinflammation and SII indicating immune exhaustion [27,28]. Metabolomic analyses link MALS to dysregulated ubiquinone biosynthesis and amino acid metabolism [29]. Importantly, the multicenter ImmunoSep RCT demonstrated that patients receiving subphenotype-specific immunotherapy (anakinra for MALS; interferon-gamma for SII) achieved a significant improvement in organ function, signaling a shift from descriptive subphenotyping toward biomarker-guided precision intervention in sepsis [30].

Static biomarker-based subphenotypes, widely applied across diverse critically ill populations [[31], [32], [33]], enable rapid immune profiling and facilitate immunotherapies. They have also been incorporated as eligibility or stratification variables in clinical trials to enhance specificity and avoid the “average treatment effect” that obscures true responders. In addition to their clinical and translational relevance, these frameworks provide mechanistic insights into immune dysregulation and may assist early prognostic stratification. However, several limitations remain, particularly the reliance on single time point measurements, which may fail to capture the dynamic immune trajectories characteristic of sepsis, and their predictive value appears largely confined to short-term outcomes. Moreover, the dependence on complex biomarker panels further restricts scalability and standardization across studies. Addressing these challenges will be critical for integrating biomarker-driven stratification into precision immunomodulatory strategies for sepsis [34].

### Dynamic subphenotypes: longitudinal analysis of static subphenotypes

**Fig. 2:**
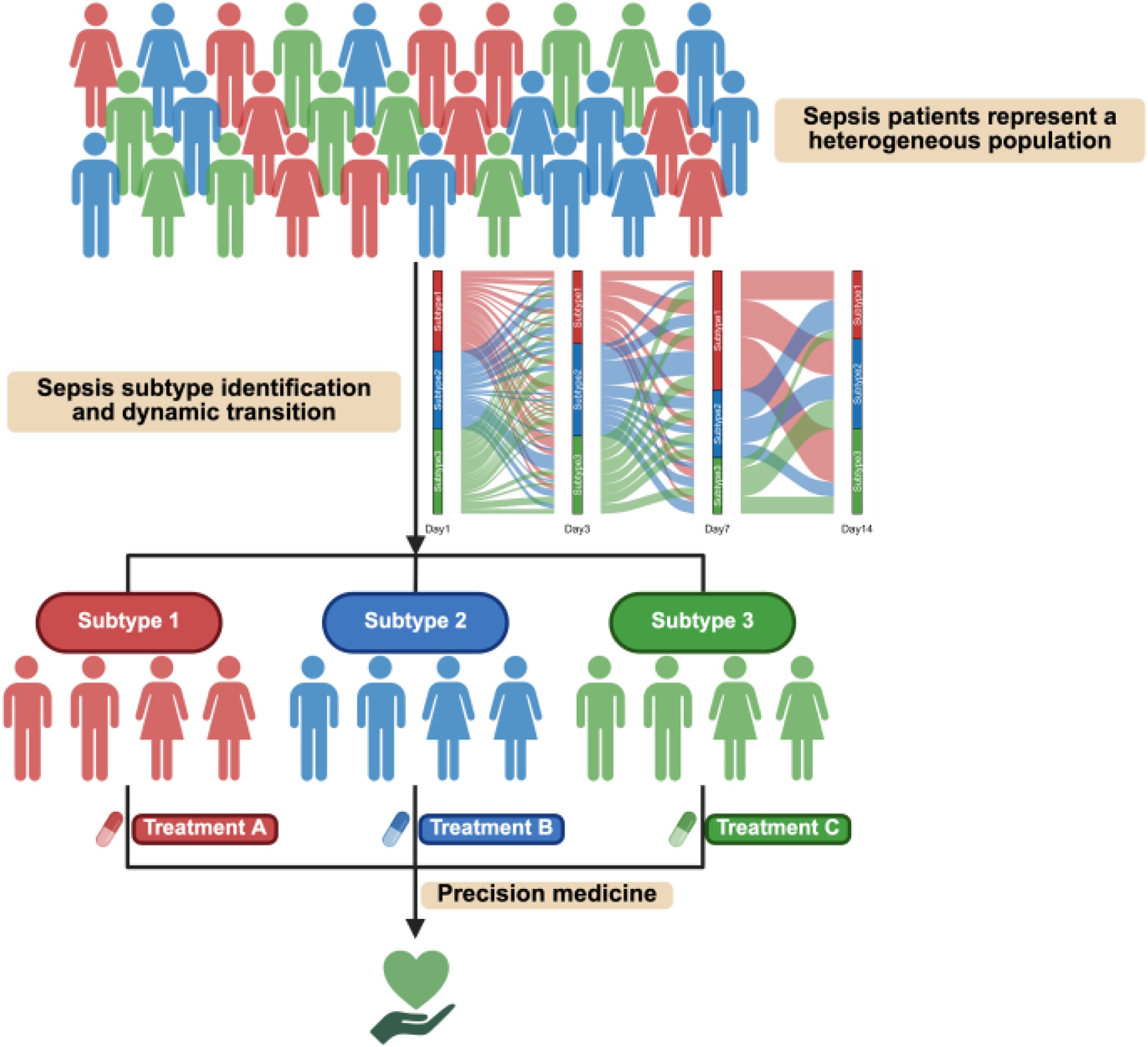
Dynamic Subtype Transitions and Precision Therapeutic Responses in Sepsis. This figure illustrates dynamic subphenotyping based on the temporal evolution of host response and organ dysfunction, enabling refined patient stratification, enhancing prognostic modeling, and supporting time-sensitive therapeutic decision-making. Figure created with BioRender.com

Traditional subphenotyping studies in sepsis have largely relied on cross-sectional data obtained at admission, limiting their capacity to reflect disease evolution. Given that sepsis represents a highly dynamic syndrome characterized by evolving pathophysiological processes, dynamic subphenotyping based on longitudinal data has emerged as a promising framework. By capturing temporal evolution in host response and organ dysfunction, this approach refines patient stratification, enhances prognostic modeling, and may inform time-sensitive therapeutic decisions (Fig. 2).

Trajectory modeling based on body temperature has demonstrated clinical feasibility for dynamic sepsis subphenotyping. In a study from a retrospective multicohort analysis, a 72 -h temperature trajectory model identified four dynamic subphenotypes: hyperthermic slow resolvers (HSR), hyperthermic fast resolvers (HFR), normothermic (NT), and hypothermic (HT), which were subsequently validated across diverse sepsis populations [[35], [36], [37], [38]]. Notably, the HT subphenotype is consistently identified as a high-risk group, characterized by immune suppression, endothelial and coagulation activation, cardiac dysfunction, and poor outcomes [[36], [37], [38]]. HSR patients display a hyperinflammatory profile with persistent fever and elevated CRP, and corticosteroid therapy has been associated with reduced likelihood of HSR membership [36,37]. Extending beyond temperature alone, multidimensional vital sign trajectories further refine risk stratification. Based on vital signs recorded during the first 8 h after hospital admission, four dynamic subphenotypes were identified, among which Group D, characterized by hypothermia, bradycardia, bradypnea, and hypotension, represents a particularly high-risk population with evidence of combined immunologic and hemodynamic impairment [39]. Treatment response heterogeneity was observed, as tocilizumab increased mortality in Groups A/B but improved survival in Group C [40], while balanced crystalloids reduced mortality in Group D [39]. Prospective studies are underway to evaluate clinical implementation and therapeutic benefit, including EHR-based subphenotyping feasibility (NCT05826223) and an RCT testing balanced crystalloids versus saline in Group D sepsis (NCT06253585) [41,42].

Apart from trajectory models, longitudinal analyses have revealed dynamic transitions between Hyper- and Hypoinflammatory subphenotypes. Persistent Hyperinflammatory states are associated with poor outcomes, whereas early transitions toward Hypoinflammatory profiles predict improved survival. [22,43]. Corticosteroid effects also appear subphenotype-dependent, with reduced mortality observed in Hyperinflammatory patients but increased mortality among Hypoinflammatory patients [22]. Complementing these findings, a cohort study of SENCA subphenotypes using fuzzy classification revealed that marginal patients were more likely to transition within 48 h, and that classification uncertainty modified treatment effects: EGDT was harmful in the core δ subphenotype but not in the marginal group [44].

Beyond the dynamic subphenotypes described above, trajectory modeling of laboratory indicators has revealed temporal heterogeneity and associations with adverse outcome [[45], [46], [47]]. These subphenotypes refine patient stratification and inform adaptive therapeutic adjustments, yet their clinical implementation faces challenges. Continuous standardized data collection is often difficult, and many patients experience frequent transitions in subphenotype membership within 48 h, which influence treatment effects [44,48]. Missing data are common for some variables, but prior studies have shown that clinical subtypes are generally robust to missingness, and multiple imputation may be used to reduce potential bias. Short observation windows and limited multi-omics validation further constrain generalizability. To overcome these challenges, simplified monitoring protocols, integration of probabilistic classification, and prospective multicenter validation are needed to ensure clinical applicability and reliable guidance for precision interventions.

## Endotypes of sepsis

With the widespread application of multi-omics technologies such as high-throughput sequencing and mass spectrometry, sepsis research is gradually shifting from descriptive subtyping toward mechanistic stratification. Omics refers to the systematic and holistic study of a specific class of biomolecules within a biological system, encompassing dimensions such as genomics, transcriptomics, proteomics, and metabolomics. The endotypes identified through omics data represent disease subgroups with defined biological mechanisms, reflecting pathological processes such as immune dysfunction, dysregulated metabolic pathways, or dysregulated cell signaling (Table 2). Compared with clinically defined subphenotypes, endotyping provides a more precise and biologically grounded foundation for advancing precision medicine in this field.

**Table 2:** Pivotal studies of endotypes in sepsis.

Endotypes	N	Clustering method	Characteristics	Differential treatment response	Classifier
Transcriptomic Endotypes					
SRS1, SRS2 [49]	371 (265 discovery, 106 validation)	Hierarchical clustering and k-means clustering	SRS1: immunosuppressed, higher serum lactate, elevated heart rate, and greater intravenous fluid requirements SRS2: relatively immunocompetent, higher prevalence of ischemic heart disease and baseline renal failure	SRS2: experienced increased mortality with hydrocortisone	Seven-gene based classifier: DYRK2, CCNB1IP1, TDRD9, ZAP70, ARL14EP, MDC1, and ADGRE3
Mars1−4 [54]	787 (306 discovery, 216 first validation, 265 second validation)	Agglomerative hierarchical clustering	Mars1: downregulation of innate and adaptive immune pathways, upregulation of metabolic pathways Mars2: upregulation of pattern recognition, cytokine, and inflammatory signaling pathways Mars3: upregulation of adaptive immune response pathways Mars4: upregulation of interferon signaling, RLR and TREM1 signaling pathways	Not tested	Gene expression ratios classifier: Mars1 score: BPGM: TAP2 Mars2 score: GADD45A: PCGF5 Mars3 score: AHNAK: PDCD10 Mars4 score: IFIT5:GLTSCR2
Inflammopathic, Adaptive, Coagulopathic [55]	1,300 (700 discovery, 600 validation)	Clustering k-means clustering and clustering Partitioning Around Medioids (PAM)	Inflammopathic: low D-dimer, high IL-6, elevated IL-1 signaling, activated pattern recognition receptors, complement cascade Adaptive: low D-dimer, low IL-6, upregulated interferon pathways, adaptive immune response Coagulopathic: high D-dimer, low IL-6, platelet degranulation, glycosaminoglycan binding, coagulation activation	Adaptive: higher probability of remaining in Adaptive endotype with anakinra Coagulopathic: reduced SRF at day 7 with anakinra	33-gene based classifier: ARG1, LCN2, LTF, OLFM4, HLA-DMB, YKT6, PDE4B, TWISTNB, BTN2A2, ZBTB33, PSMB9, CAMK4, TMEM19, SLC12A7, TP53BP1, PLEKHO1, SLC25A22, FRS2, GADD45A, CD24, S100A12, STX1A, KCNMB4, CRISP2, HTRA1, PPL, RHBDF2, ZCCHC4, DDX6, SENP5, RAPGEF1, DTX2, RELB
USE-1/USE-2 [62]	355 (243 discovery, 112 validation)	Hierarchical clustering on principal components	USE‐1: relatively low levels of inflammation; less severe illness, with lower mortality USE‐2: neutrophil-dominated innate immune activation and suppression of adaptive lymphocyte responses; higher lactate levels, impaired functional status, and elevated physiological severity scores, with higher mortality	Not tested	13-gene random forest classifier: CD55, IFNGR2, PSTPIP2, SDCBP, NFE2L2, ACTN1, LRP10, PICALM, USP32, OXNAD1, PRMT7, ZBTB40, and ZNF783
Proteomic Endotypes					
USS-1, USS-2 [65]	495 (242 discovery, 253 validation)	Agglomerative hierarchical clustering and k-means clustering	USS-1: relatively low levels of inflammation; less severe illness, with lower mortality USS-2: proinflammatory innate response, T-cell dysregulation, Endothelial dysfunction; more severe physiological derangement, higher prevalence of HIV and active TB	Not tested	5-protein classifier: CD40, PGF, TIMP1, CD46, and CST3
Metabolomic Endotypes					
Cluster 1−3 [61]	643 patients (470 derivation, 173 validation) with 1,895 total samples (1,402 derivation, 493 validation)	K-means clustering	Cluster 1: the high-metabolite and low-mortality cluster Cluster 2: the low-metabolite cluster Cluster 3: the low-lysophospholipid and high-triglyceride cluster	Cluster 1−3: showed no differential response to trial interventions (Levosimendan vs. placebo; norepinephrine vs. vasopressin; hydrocortisone vs. placebo) across baseline metabolic profiles	None reported

### Transcriptomic endotypes

To investigate the molecular characteristics of immune dysfunction in septic patients, multiple studies have leveraged whole-genome transcriptomic data from peripheral blood mononuclear cells, identifying immune endotypes distinguished by differential inflammatory signaling. In a cohort of patients with sepsis caused by community-acquired pneumonia, two transcriptomic sepsis response signatures (SRS1 and SRS2) were initially identified [49]. SRS1, characterized by T-cell exhaustion, endotoxin tolerance, and HLA-II downregulation, is associated with increased mortality. These endotypes have been validated across bacterial and viral sepsis cohorts, with machine learning models enabling classification [[50], [51], [52]]. Treatment heterogeneity is evident, with hydrocortisone increased mortality in SRS2 but not in SRS1 patients [49,53].

Distinct from these two-endotype frameworks, the Molecular Diagnosis and Risk Stratification of Sepsis (MARS) consortium identified four transcriptomic endotypes (Mars1–4) in sepsis, with Mars1 exhibiting marked immunosuppression and highest mortality, Mars2 reflecting NF-κB/IL-6–driven hyperinflammation, and Mars4 upregulating interferon and RLR pathways [54]. Separately, three transcriptomic endotypes (Inflammopathic, Adaptive, and Coagulopathic) were defined across multiple cohort, with parsimonious gene classifiers subsequently developed; the Coagulopathic endotype was associated with Coagulopathic endotype associated with aberrant coagulation and the Inflammopathic endotype with proinflammatory signaling [[55], [56], [57]]. Post hoc analysis of SAVE-MORE showed anakinra stabilized the Adaptive endotype and reduced severe respiratory failure in Coagulopathic patients, highlighting endotypes as dynamic therapeutic strata [58].

### Proteomic and metabolomic endotypes

While transcriptomic studies have highlighted immune heterogeneity, proteomic and metabolomic analyses reveal additional layers of dysregulation, revealing complementary axes of immune and metabolic variation.

Through plasma proteomic profiling of sepsis patients, three subgroups, referred to as sepsis plasma proteome-based clusters (SPC1–3), were defined, showing distinct clinical and biological characteristics [59]. SPC1 exhibited the most severe disease, with higher Sequential Organ Failure Assessment (SOFA) scores and mortality, alongside elevated immunoglobulins, apolipoproteins, extracellular matrix proteins, and active immune pathways, but reduced lipoprotein metabolism. SPC2 represented an intermediate phenotype with moderate immune activity and lower B cell signaling. SPC3 encompassed younger patients with reduced cytokine signaling and milder disease. Dynamic transitions, mainly from SPC1 to SPC3, indicate that plasma proteomics–driven classification reflects both disease severity and recovery, supporting its utility for mechanistic patient classification and therapeutic targeting.

Increasing evidence suggests that sepsis involves not only immune dysregulation but also significant metabolic disturbances [60]. In recent years, metabolomics has been increasingly incorporated into the study of sepsis endotypes, deepening our understanding of immune heterogeneity from a metabolic perspective and aiding the development of biologically and clinically meaningful classification models. In a study analyzing a septic shock cohort, three metabolomic endotypes were defined, temporal trajectories in relation to patient outcomes were assessed, and an elastic net classifier was constructed [61]. Cluster 1 exhibited broadly elevated metabolites, particularly lipids, and evolved over time to the dominant trajectory. Clusters 2 and 3 were associated with persistent metabolic suppression and increased organ dysfunction, with Cluster 3 showing a distinct signature of elevated triglycerides, and Cluster 2 characterized by relative enrichment of small molecules and carnitines. Although these metabolomic endotypes did not appear to modify treatment responsiveness, their dynamic evolution underscores the importance of incorporating temporal stratification into sepsis research and therapeutic trial design.

Other than these studies, multiple studies have identified clinically relevant endotypes in both adult and pediatric sepsis patients based on transcriptomic [[62], [63], [64]], proteomic [65,66], and metabolomic profiles [67]. These endotypes capture biological heterogeneity in inflammatory and immune responses, metabolic dysregulation, and susceptibility to organ dysfunction, providing opportunities for early risk stratification, optimization of intervention timing, and individualized treatment decisions. Emerging evidence indicates that sepsis endotypes represent temporally dynamic biological states rather than static categories, evolving throughout the disease course and in response to therapeutic interventions [68]. Recent longitudinal analyses indicate that only a minority of patients retain their initial endotype after one week [58,59,61], reinforcing the need for continuous molecular reassessment and time-adaptive immunomodulatory strategies. Despite their potential, clinical translation is challenged by technical complexity and cost, as well as confounding factors including nutrition, concomitant medications, and heterogeneity in patient management. Future efforts should focus on developing simplified classifiers based on a minimal biomarker set, extending analyses to special populations such as immunocompromised or transplanted patients, integrating multi-omic and clinical data for refined patient stratification, and evaluating whether real-time endotype monitoring can guide adaptive immunomodulatory interventions.

## Integrating Multidimensional Data in Sepsis: Bridging Clinical Features and Biological Mechanisms

**Fig. 3:**
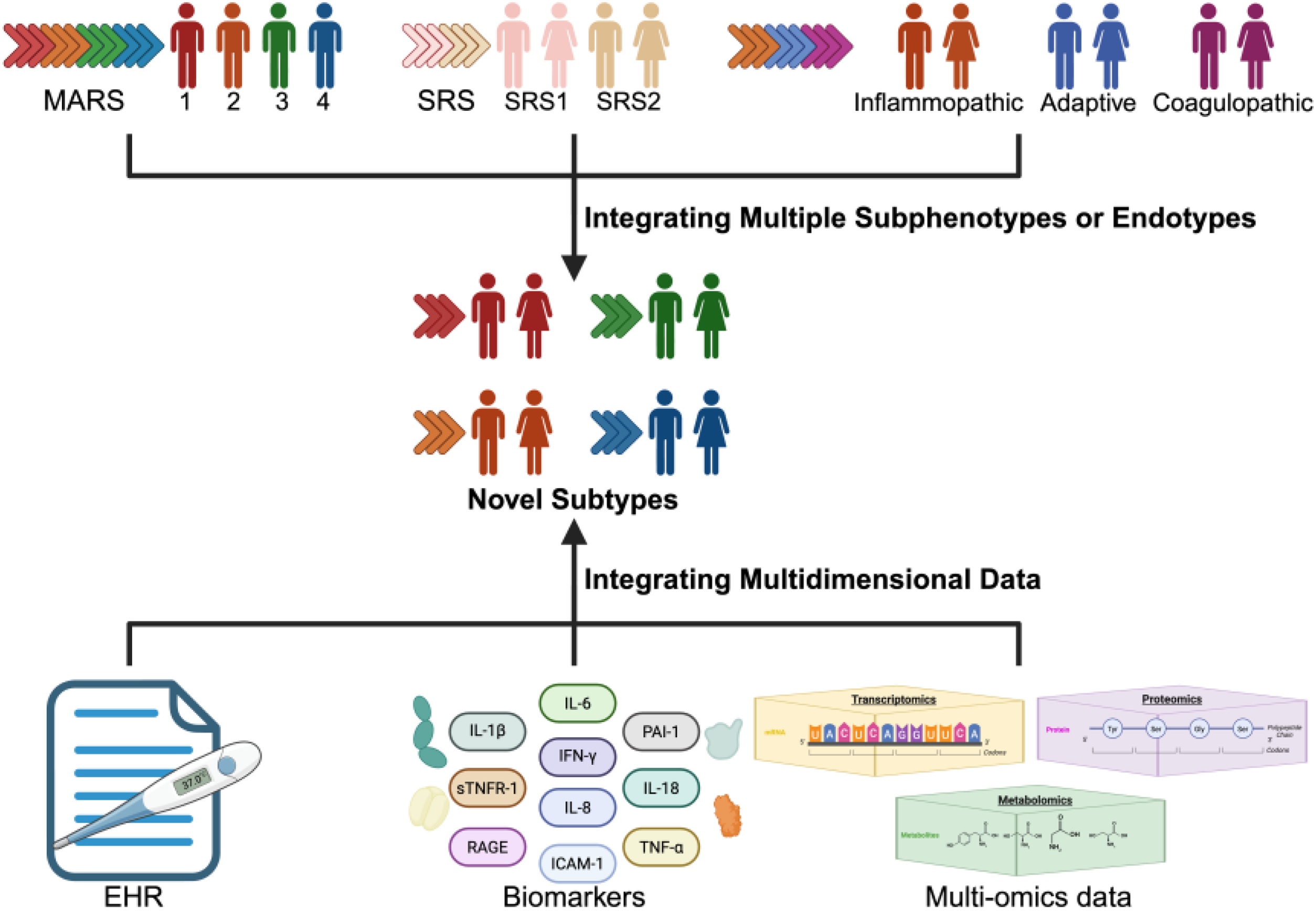
Integrated analytical framework for sepsis subtyping. This Figure illustrates how multiple subphenotypes or endotypes can be integrated, and how multidimensional data can be combined to bridge clinical manifestations with underlying biological mechanisms, ultimately supporting optimized classification and precision therapeutic strategies. Figure created with BioRender.com

Sepsis is a highly heterogeneous clinical syndrome characterized by diverse clinical features and complex pathophysiological changes, resulting in marked heterogeneity of treatment response [4]. Although substantial progress has been made in identifying various subphenotypes and endotypes of sepsis, subphenotypes derived from clinical features and biomarkers primarily capture treatment response heterogeneity with strong clinical relevance, whereas endotypes reveal underlying mechanisms but are less directly applicable to bedside therapeutic decision-making. Importantly, even within identical datasets, different clustering methods may yield inconsistent subtypes that often show poor concordance, underscoring the methodological dependence of subtype identification and the need for cross-validation across independent cohorts. In addition, most precision treatment analyses based on current subtyping frameworks demonstrate potential therapeutic stratification, the majority of evidence stems from retrospective analyses, in which benefit in one subgroup may correspond to harm in another. Researchers have increasingly adopted strategies that integrate multidimensional data, combining subphenotypes, endotypes, and their temporal dynamics to bridge clinical features with biological mechanisms and optimize sepsis classification and therapeutic strategies (Fig. 3).

While numerous molecular endotypes have been proposed in sepsis, the biological overlap between them remains elusive. A systematic comparison of the SENECA, Hyper-/Hypoinflammatory, MARS, and SRS classification frameworks revealed limited concordance across systems [69], suggesting that each captures distinct but only partially overlapping dimensions of sepsis. Accordingly, harmonizing these subgrouping strategies is essential to enable their coherent integration into future clinical practice and to ultimately advance toward a consensus model of sepsis [70,71]. Addressing the absence of a standardized framework of sepsis, a Markov clustering approach integrating SRS, Mars, and inflammation-related endotypes identified three consensus transcriptomic subtypes (CTS1-3) [72]. Notably, CTS1 is defined by a profound immunopathology characterized by systemic hyperinflammation and extensive endothelial activation, which correlates with persistent clinical instability and poor prognosis. While CTS2 and CTS3 are defined by distinct signatures of heme metabolism and coagulation dysfunction and interferon signaling and lymphocyte enrichment respectively, the biological validity of these subtypes underscores the complex heterogeneity of sepsis.

Nevertheless, classification strategies relying on a single omics modality often fail to fully capture the complex heterogeneity of patients with sepsis. The combined SRS–SPC framework revealed that dual SRS1–SPC1 classification predicted the poorest prognosis [59], highlighting the translational potential of integrating distinct endotyping frameworks for precision stratification in sepsis. In Ugandan cohorts, researchers identified transcriptomic endotypes (USE-1/USE-2), and proteomic endotypes (USS-1/USS-2) [62,65]. USE-2 and USS-2 were both characterized by proinflammatory innate immune activation and were associated with severe illness and increased mortality, whereas USE-1 and USS-1 reflected relatively balanced immune responses. Despite shared biological themes, the concordance between USE and USS classifications was low, as transcriptomics reflects upstream regulatory processes of biological mechanisms while proteomics captures the functional states of downstream effector molecules. These findings indicate that different modalities emphasize distinct yet complementary dimensions when capturing sepsis heterogeneity. Accordingly, the integration of multimodal data holds promise not only for bridging the gap between clinical features and pathophysiological mechanisms, and between upstream signaling and downstream effector molecules, but also for improving the stability and clinical generalizability of sepsis subtyping.

In this context, multimodal machine learning has garnered increasing attention. As a critical subfield of machine learning, multimodal learning aims to develop and train models capable of leveraging multiple data types—such as EHR, clinical laboratory results, plasma biomarkers, imaging data, and multi-omics profiles—by capturing the intrinsic relationships across modalities to improve predictive performance [73]. In oncology, this strategy has been widely applied to tasks such as patient risk stratification, prognosis assessment, and treatment response prediction, enhancing model generalizability while enabling mechanistic interpretation across clinical and molecular domains [[74], [75], [76]]. However, current applications of multimodal machine learning in critical care have largely focused on early sepsis detection and outcome prediction, while its utility in sepsis subtyping remains relatively underexplored [[77], [78], [79]].

Taken together, the integrated analysis of subphenotypes and endotypes provides strong support for precision diagnosis, targeted therapy, and stratified intervention in sepsis, representing a key direction for future research and clinical translation. Multimodal machine learning offers a technical framework for integrating subphenotypes and endotypes, with the potential to bridge clinical features and molecular mechanisms. By combining diverse data modalities, this approach enables a more comprehensive characterization of sepsis heterogeneity, thereby enhancing the scientific basis of patient stratification and the feasibility of targeted interventions, and ultimately facilitating the translation of precision medicine into critical care.

## Conclusion and perspectives: pathways and challenges toward precision medicine in sepsis

Sepsis remains a major global healthcare challenge and a leading contributor to global health burden. Despite increasing efforts to classify sepsis into subphenotypes and endotypes, and to integrate multimodal data for precision therapy, sepsis subtype research remains in an exploratory stage and faces persistent challenges. These challenges include the absence of harmonized classification criteria, significant methodological heterogeneity, limited generalizability of predictive models, high cost and complexity of multi-omics assays, inadequate real-time analytics in clinical workflows, and limited therapeutic actionability. Collectively, these limitations hinder the translation of subtype research into interventions that meaningfully impact patient outcomes.

Going forward, research should focus on the development of multimodal, longitudinal consensus sepsis frameworks and prospectively evaluate heterogeneity of treatment effects across sepsis subtypes to identify patient groups that may benefit or be harmed by specific interventions. Such frameworks should aim to capture both upstream regulatory mechanisms and downstream effector states, thereby providing mechanistic insights and guiding rational therapeutic stratification. The development of POCT platforms capable of rapidly identifying patient subtypes, along with the validation of their generalizability and robustness, is essential. Operationally, classifiers could be simplified into parsimonious biomarker panels integrated into EHR systems, enabling automated subtype assignment within the first hours of ICU admission and dynamic reclassification as clinical parameters evolve. This approach may facilitate timely treatment adjustments and reduce exposure to ineffective or potentially harmful therapies. Furthermore, future research should ensure global representativeness by incorporating data from low- and middle-income countries, and leverage multicenter longitudinal multimodal datasets to track dynamic biological trajectories over time, thereby enabling the development of adaptive treatment algorithms applicable across diverse clinical settings worldwide.

## Authors' contributions

Z.G. contributed to the conceptualization of the review, performed the literature search and data analysis, interpreted the findings, and drafted the manuscript. Z.G. and W.X. contributed to the graphic depiction and data analyses of the manuscript. C.C., L.J., L.Si., and W.P. checked the data for the article. L.So., X.H., and X.J. provided critical revisions and valuable suggestions to improve the manuscript. All authors contributed to the article and approved the submitted version.

## Consent for publication

Not applicable.

## Ethics approval and consent to participate

Not applicable.

## Fundings

Funding was provided by the Noncommunicable Chronic Diseases National Science and Technology Major Project (No. 2023ZD0506506to J.F.X.). This work was also supported by the National Natural Science Foundation of China (No. 82272210; No.82572482 to J.F.X., No.82572516 to X.J.W.), the Yunnan Province Science and Technology commission (No. 202305AF150186 to J.F.X.), and the Jiangsu Province High-Level Hospital Construction Funds of Zhongda Hospital, School of Medicine, Southeast University (CZXM-GSP-KY-JCYJ06 to J.F.X.).

## Availability of data and material

Not applicable.

## Declaration of competing interest

The authors declare that they have no competing interests.
